# 1275‐nm Photobiomodulation Alleviates Brain Drainage Impairment as a Promising Therapeutic Strategy for Aging‐Related Neurological Decline

**DOI:** 10.1111/acel.70261

**Published:** 2025-10-15

**Authors:** Hao Lin, Shaojun Liu, Qihang Yang, Junming Li, Jue Wang, Oxana Semyachkina‐Glushkovskaya, Dongyu Li, Tingting Yu, Dan Zhu

**Affiliations:** ^1^ MOE Key Laboratory for Biomedical Photonics, Wuhan National Laboratory for Optoelectronics ‐ Advanced Biomedical Imaging Facility Huazhong University of Science and Technology Wuhan Hubei China; ^2^ Department of Hematology, Tongji Hospital of Tongji Medical College Huazhong University of Science and Technology Wuhan Hubei China; ^3^ Saratov State University Saratov Russia; ^4^ School of Optical Electronic Information Huazhong University of Science and Technology Wuhan Hubei China

**Keywords:** aging, cognitive function, drainage, meningeal lymphatic vessel, neuroinflammation, oxidative stress, photobiomodulation

## Abstract

Aging imposes a significant socioeconomic and healthcare burden worldwide, while effective therapy is still lacking. Impaired brain drainage and excessive accumulation of metabolites and toxins such as advanced glycation end products (AGEs) are characteristics of aging that contribute to the development of neurological disorders. Recent discoveries have highlighted the role of meningeal lymphatic vessels (MLVs) in the clearance of toxic metabolites, cells, tumors, and viruses from the brain, positioning them as significant targets for the treatment of various brain diseases. In this study, we demonstrate that noninvasive 1275‐nm photobiomodulation (PBM) effectively improves brain drainage and promotes lymphatic clearance of AGEs in a D‐galactose‐induced aging model (AM) in male mice, while being safe due to its minimal thermal effects. These improvements are associated with nitric oxide release‐mediated dilation of MLVs. PBM can also effectively ameliorate redox imbalance, neuroinflammation, and neuronal damage, as well as improve spatial learning ability and short‐term recognition memory in AM mice. These findings introduce a promising and easily accessible strategy for nonpharmacological phototherapy of meningeal brain drainage and neurological decline in individuals with aging and aging‐related neurodegenerative diseases, offering high potential for rapid implementation into routine clinical practice.

## Graphical abstract

Noninvasive 1275‐nm photobiomodulation improves brain drainage and promotes lymphatic clearance of advanced glycation end products (AGEs) in an aging model (AM) in male mice. These effects are associated with nitric oxide release‐mediated dilation of meningeal lymphatic vessels (MLVs). PBM can also ameliorate redox imbalance, neuroinflammation, neuronal damage, and reduce cognitive dysfunction in AM mice.

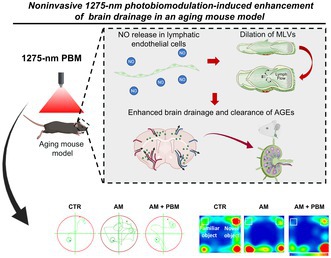

**Abbreviations**
AD: Alzheimer's disease
AGEs: advanced glycosylation end products
AM: aging model
Aβ: amyloid beta
CBF: cerebral blood flow
CSF: cerebrospinal fluid
CTR: control
dcLNs: deep cervical lymph nodes
D‐gal: D‐galactose
DI: discrimination index
EBD: Evans blue dye
ELISA: enzyme‐linked immunosorbent assay
FITCD: fluorescein isothiocyanate‐dextran
LECs: lymphatic endothelial cells
MBP: myelin basic protein
MDA: malondialdehyde
MLVs: meningeal lymphatic vessels
MWM: Morris water maze
NO: nitric oxide
NOR: novel object recognition
PBM: photobiomodulation
PBS: phosphate buffered saline
PD: Parkinson's disease
PSS: petrosquamosal sinus
ROS: reactive oxygen species
SOD: superoxide dismutase

## 1 Introduction

The global aging population is expanding at an unprecedented rate, with the number of individuals aged 60 and above projected to reach 2 billion by 2050 (Bloom 2011). As a result, the incidence of diseases linked to age‐related neurological disorders, such as Alzheimer's disease (AD), Parkinson's disease (PD), and multiple sclerosis, has risen significantly (Hou et al. 2019). Despite significant efforts in the development of new pharmacological strategies and novel therapeutics such as stem cell treatments and plasma transfusion therapeutics for improving aging‐related neurological decline, there is a lack of effective, safe, and easily accessible therapies that can be readily applied in clinical practice.

Aging is typically linked to the accumulation of metabolites and toxins, along with impairments in brain drainage (Da Mesquita et al. 2018). Advanced glycation end products (AGEs) are a complex and heterogeneous group that accumulate significantly in the aging brain via the nonenzymatic Maillard reaction, the polyol pathway, and lipid peroxidation (Chaudhuri et al. 2018; Ott et al. 2014). AGEs are considered potential drivers of aging (Chaudhuri et al. 2018). Increasing evidence suggests that AGEs can promote redox imbalance, amyloid beta (Aβ) production, mitochondrial dysfunction, chronic inflammation, and neuronal apoptosis, and these changes significantly contribute to aging‐related cognitive decline (Chaudhuri et al. 2018; Dei et al. 2002; Ko et al. 2010; Tan et al. 2015). Direct evidence demonstrates that the injection of AGEs promotes Aβ production in cells; moreover, the injection of AGEs into rat brain tissue leads to redox imbalance, elevates NF‐κB expression, and causes cognitive impairment (Ko et al. 2010; Tan et al. 2015). Strategies aimed at reducing AGEs accumulation represent a promising therapeutic approach for improving aging‐related abnormalities (Aydın et al. 2017; Byun et al. 2017; Lu et al. 2010; Tan et al. 2015).

In recent years, it has been established that the meningeal lymphatic vessels (MLVs) are “tunnels” that facilitate brain drainage and removal of metabolites, cells, cytokines, and viruses from the brain, thereby ensuring the maintenance of homeostasis of the central nervous system (Chen et al. 2020; Da Mesquita et al. 2018, 2021; Dupont et al. 2020; Li, Qi, et al. 2022; Li, Liu, et al. 2023; Semyachkina‐Glushkovskaya, Sokolovski, et al. 2023; Song et al. 2020). It is logical to assume that MLVs are involved in the clearance of other toxic metabolites, such as AGEs, which, however, have not yet been studied. With age, morphological changes in MLVs are observed, accompanied by a significant impairment in brain drainage, which explains the deposition of Aβ and toxic metabolites like AGEs in the tissues of the aging brain (Da Mesquita et al. 2018). A substantial number of pilot studies have shown that increasing the activity of MLVs by stimulation of lymphangiogenesis improves the therapy of aging, AD, and brain tumors (Da Mesquita et al. 2018, 2021; Hu et al. 2020; Song et al. 2020).

It is important to note that the proposed single pharmacological method for stimulating MLVs by injection of the vascular endothelial growth factor C into the cisterna magna is invasive and therefore limited in its widespread use in clinical practice (Da Mesquita et al. 2021). Therefore, nonpharmacological methods of photobiomodulation (PBM) targeting MLVs are both relevant and in high demand. MLVs are located on the surface of the brain in its meninges. Therefore, they are the ideal targets for light. It is well known that the tissue light scattering decreases with increasing wavelength (Genina et al. 2019). A growing number of studies indicate that near‐infrared PBM can stimulate MLVs and increase their lymphatic drainage properties, exerting therapeutic effects in the development of AD (Li, Lin, et al. 2023; Salehpour et al. 2022; Wang, Yan, et al. 2024), glioma (Semyachkina‐Glushkovskaya, Sokolovski, et al. 2023), and diabetic brain damage (Liu et al. 2023). Improved MLVs drainage may exert beneficial effects on downstream glial reactivity in the hippocampus and cognitive function by clearing proinflammatory cytokines (Dong et al. 2024; Hsu et al. 2021; Liu et al. 2023).

Based on the above, we hypothesized that near‐infrared (1275 nm) PBM may be a promising method to improve brain drainage function and stimulate the elimination of AGEs in the aging brain. To test this hypothesis, we used a typical high‐dose D‐galactose (D‐gal)‐induced mouse aging model (AM), which recapitulates complex neuropathological changes associated with aging and neurodegenerative diseases, such as AGEs formation, Aβ deposition, neuroinflammation, neurodegeneration, and oxidative stress (Pantiya et al. 2023; Shwe et al. 2018). We investigated the changes in the MLVs network and their drainage function in AM mice, and explored the effects of 1275‐nm PBM on these abnormalities, as well as on aging‐related neuropathological changes. We found that PBM is a safe and effective approach for enhancing meningeal brain drainage and the clearance of excessive AGEs in AM mice. PBM can also effectively ameliorate redox imbalance, neuroinflammation, and neuronal damage, as well as improve spatial learning ability and short‐term recognition memory in AM mice. Our findings highlight the potential of 1275‐nm PBM as a promising and nonpharmacological therapeutic strategy for improving aging and aging‐related neurodegenerative disorders.

## 2 Materials and Methods

### 2.1 Animals

Adult male C57BL/6 and Cx3cr1‐GFP mice, aged ~3 months and weighing 22–30 g, were housed in an animal room under a controlled temperature of 23°C and a 12 h light–dark cycle, with access to water and food ad libitum. C57BL/6 mice were purchased from the Liaoning Changsheng Biotech Co. Ltd. (Benxi, China). Cx3cr1‐GFP mice (B6.129P‐*Cx3cr1* ^*tm1Litt*^/J, Stock No. 005582) were from the Jackson Laboratory (Bar Harbor, ME, USA). All animals were approved by the Experimental Animal Management Ordinance of Hubei Province, P. R. China, and the study was carried out in accordance with the “Guide for the Care and Use of Laboratory Animals.” The mice were randomly assigned to one of three groups: control (CTR), AM, or AM+PBM.

In this study, we used the D‐gal‐induced aging model, a well‐established model in aging research (Azman and Zakaria 2019; Lee et al. 2020; Liang et al. 2022; Wang, Yu, et al. 2024; Wu et al. 2022), distinct from diabetes models due to the absence of sustained fasting hyperglycemia (Dong et al. 2017). To establish this model, mice received daily intraperitoneal injections of 500 mg/kg D‐gal (G0750, Sigma, USA). As a control, mice in the CTR group received daily intraperitoneal injections of 0.2 mL normal saline (0.9% NaCl) for the same duration (Azman and Zakaria 2019). The underlying mechanisms of D‐gal‐induced aging involve: (1) galactitol accumulation via aldose reductase, inducing osmotic stress and mitochondrial dysfunction; (2) hydrogen peroxide generation through oxidation, depleting superoxide dismutase (SOD) and disrupting redox homeostasis; and (3) nonenzymatic glycation that forms AGEs (Azman and Zakaria 2019).

### 2.2 PBM Treatment

A commercially available laser diode (MDL‐H1275, Changchun New Industries Optoelectronics Tech. Co. Ltd., China) emitting at 1275 nm was used for noninvasive irradiation sessions. The laser diode was connected to a fiber and beam expander, emitting a uniform circular light spot, and was fixed vertically downwards.

For in vivo irradiation, the laser beam expander was fixed vertically downwards in a self‐designed cylindrical apparatus that allowed mice to move freely (Figure S1A). Single PBM session comprised three cycles: 17‐min irradiation followed by a 5‐min pause, totaling 61 min per day (Figure S1B). In this study, four laser power densities on the shaved scalp surface were used: 5, 10, 20, and 40 mW/cm^2^, corresponding to energy densities of approximately 15, 30, 60, and 120 J/cm^2^. With a measured transmittance of approximately 33% for the 1275‐nm laser through the cranial bone and scalp of 5‐month‐old C57BL/6 mice, the laser power densities reaching the cortical surface were estimated to be 1.6, 3.2, 6.4, and 12.8 mW/cm^2^, corresponding to energy densities of approximately 5, 10, 20, and 40 J/cm^2^. After an 8‐week D‐gal treatment, the AM mice received PBM treatments daily for 2 weeks (PBM course), with total cumulative doses of 210, 420, 840, and 1680 J/cm^2^ on the scalp.

For in vitro irradiation, the laser beam spot was adjusted to cover the cell culture well. The cells were exposed to the laser for 17 min, receiving an approximately dose of 3.3 J/cm^2^ (power density: 3.2 mW/cm^2^).

The change in the cortical surface temperature induced by laser exposure was monitored using a thermocouple system (YET‐620L, CAIPUSEN, China). Specifically, the medial part of the left temporal muscle was detached from the skull bone. Then, a minor burr hole was drilled on the temporal bone, through which a flexible thermocouple probe was inserted to the cortical surface. A thermocouple data logger was used to record the temperature changes throughout the single PBM session.

### 2.3 Morris Water Maze (MWM) Test

The MWM test was performed to assess the spatial learning and memory abilities (Wang, Yan, et al. 2024). Briefly, a circular water tank measuring 120 cm in diameter and 50 cm in height was filled with water to a depth of 30 cm. White food additives were added to make the water opaque, and an inconspicuous heater was used to keep the water temperature constant (22°C ± 1°C). A transparent, circular survival platform (10 cm in diameter) was hidden 1 cm below the water surface in one quadrant of the tank. During the training sessions, the mice received four 1‐min training trials per day across different quadrants for five consecutive days. A probe trial (1 min) was conducted 24 h after the training (Day 6) with the hidden platform removed. The time taken to find the survival platform (escape latency), and the escape trajectory were recorded and analyzed using a computerized tracking system (Supermaze, Shanghai Xinruan Information Tech. Co. Ltd., China). The swimming speed was calculated by dividing the cumulative distance by the escape latency.

### 2.4 Novel Object Recognition (NOR) Test

NOR test was performed to measure the short‐term memory ability of mice, as previously described (Li, Lin, et al. 2023). During the habituation session, the mice were placed in an empty open field and allowed to freely explore for 10 min. After 24 h, two identical nontoxic objects were placed in opposite and symmetrical corners of the open field. Each mouse was then released into the open field and allowed free exploration for a 5‐min period. After a 6‐h period, one of the familiar objects was replaced by a novel object in the test session. The mice were returned to explore the open field for an additional 5 min to test the preference for these two objects. A discrimination index (DI), calculated as follows, was used to reflect the cognitive function of the mice:

$$\mathrm{DI}=\left(T_{\text{novel}}-T_{\text{familiar}}\right)/\left(T_{\text{novel}}+T_{\text{familiar}}\right)$$

where *T*_novel_ and *T*_familiar_ indicate the exploration time for the novel and familiar objects during test session, respectively. The raw positional data from the training and testing sessions, along with subsequent analyses of total distance traveled, and object exploration preference were obtained using a computerized tracking system (Supermaze, Shanghai Xinruan Information Tech. Co. Ltd., China). The average locomotor velocity was determined by dividing the total distance traveled by the total recording time. Note: Prior to each habituation, training, and test session, the arena and objects were thoroughly sprayed with 75% ethanol to eliminate residual odors and allowed to air dry completely before proceeding.

### 2.5 Tissue Preparation

For immunofluorescence, the deep cervical lymph nodes (dcLNs) of the mice were obtained under deep anesthesia, and the meninges and brains were collected after cardiac perfusion. Subsequently, the obtained tissues were fixed in 4% paraformaldehyde solution at 4°C overnight, followed by a phosphate‐buffered saline (PBS) rinse. Then, the brains and 2% agarose‐embedded dcLNs were sliced into 100‐μm‐thick sections by a vibratome (VT1000; Leica, Germany).

For Western blot and biochemical assay, the brains and meninges of mice were quickly and carefully dissected under deep anesthesia. The tissues were then homogenized in RIPA buffer for Western blot or in PBS for biochemical assays, with both buffers containing phosphatase and protease inhibitor cocktails. This was followed by centrifugation at 13,500 × *g* at 4°C for 15 min. The supernatants were collected and stored at −80°C until use.

### 2.6 Immunofluorescence Staining

Immunofluorescence staining was performed as described previously (Li, Lin, et al. 2023; Liu et al. 2023). In brief, the brain slices, dcLN slices, and meninges were incubated in blocking solution (a mixture of 0.2% Triton X‐100 and 10% normal goat serum in PBS) for 1, 1, and 4 h at room temperature, respectively. Subsequently, the samples were incubated overnight at room temperature with appropriate primary antibodies in 0.2% Triton X‐100. The following antibodies were used (see Table S1): anti‐Lyve‐1 (1:500; FAB2125G, R&D Systems, USA), MBP (1:500, 15089‐1‐AP, Proteintech, China), GFAP (1:1000, MAB360, EMD Millipore, Germany), and AGEs (1:250, ab23722, Abcam, UK). Afterward, the samples were washed with 0.2% Triton X‐100 for at least 30 min and then incubated with appropriate fluorescent‐labeled secondary antibodies (Alexa Fluor 555 goat antirabbit IgG (H + L), 1:500, Invitrogen, USA; Alexa Fluor 555 goat antimouse IgG (H + L), 1:500, Invitrogen, USA; Alexa Fluor 647 goat antirabbit IgG (H + L), 1:500, Invitrogen, USA) for 4 h at room temperature. Subsequently, the brain slices were mounted with DAPI dye (1:1000; D1306, Invitrogen, USA) for 10 min, followed by washing for at least 30 min. Fluorescent images were acquired using a confocal laser scanning microscope (LSM 710, Zeiss, Germany) equipped with 20× and 63× objectives; z‐stacks were collected at 2 μm intervals.

### 2.7 Brain Drainage Assay

For evaluation of brain drainage ability in vitro, an amount of 5 μL of fluorescein isothiocyanate‐dextran (FITCD, 1%, 70 kDa, Sigma, USA) was injected into the cisterna magna of the anesthetized mice at a rate of 0.1 μL per min using a microinjector. Following complete injection, the needle was carefully removed and the laser was turned on. Following the PBM session, the mice were sacrificed, with the brains and dcLNs harvested. A confocal laser scanning microscope (LSM 710, Zeiss, Germany) was used to capture the FITCD distribution of the basal and dorsal brain, as well as dcLNs. Additionally, to monitor the infusion of the FITCD from the cisterna magna into the brain parenchyma, the brains were fixed in 4% paraformaldehyde solution at 4°C overnight. Subsequently, the brains were sectioned into six 100‐μm‐thick slices using a vibratome (VT1000, Leica, Germany) and then imaged with a confocal laser scanning microscope (LSM 710, Zeiss, Germany).

For evaluation of the brain lymphatic clearance capacity in vivo. A small burr hole was made over the right lateral ventricle (AP = −0.5 mm, ML = −1.06 mm) in the anesthetized mice. 5 μL of Evans blue dye (EBD, 1%, Sigma, USA) was injected into the right lateral ventricle at a speed of 0.5 μL per min to a depth of 2.5 mm using a microinjector. The aggregation of EBD in the dcLNs was imaged by a stereo fluorescence microscope (Axio Zoom V16, Carl Zeiss, Germany).

### 2.8 Laser Speckle Contrast Imaging

The skulls of mice were cleared, allowing for the observation of cortical blood vessels through a long‐term skull optical clearing window (Li, Hu, et al. 2022). After a 3‐day recovery period, the mice were anesthetized with 1% isoflurane (in N_2_/O_2_‐70:30) with a flow of 1 L/min, with their body temperature maintained at 37°C using a heating pad. A laser speckle contrast imaging system (RWD Life Science, China) was employed to measure cerebral blood flow (CBF) in the mice. The transmittance of the 1275‐nm PBM through the cleared skull is approximately 79%; the PBM doses (single session) at the cleared skull and brain surface were 12.7 J/cm^2^ and 10 J/cm^2^, respectively.

### 2.9 TUNEL Assay

A commercial TUNEL assay kit (E‐CK‐A320, Elabscience, China) was utilized to detect cellular apoptosis. According to the manufacturer's protocol, slices were sequentially subjected to protease K solution digestion (1%, 20 min, 37°C), followed by PBS washing, equilibration buffer incubation (20 min, 37°C), terminal deoxynucleotidyl transferase working solution incubation (2 h, 37°C), and a final PBS washing. Afterward, the fluorescent images were captured by a fluorescence microscope (Ni‐E, Nikon, Japan).

### 2.10 L‐NAME Treatment

L‐NAME, a blocker of nitric oxide (NO) synthase, was administered following the procedures outlined in previous publications (Liu et al. 2023; Trujillo et al. 2017). In brief, L‐NAME (N5751, Sigma, USA) was reconstituted according to the manufacturer's instructions. Subsequently, 5 μL (100 mg/mL) of L‐NAME was injected into the cisterna magna of the anesthetized mouse at a speed of 1 μL per min. 4 h later, the mouse received single PBM treatment.

### 2.11 Inflammatory Cytokines Assay

Commercial enzyme‐linked immunosorbent assay (ELISA) kits (ABclonal, China) were used to measure the levels of IL‐1β, IL‐4, IL‐6, and IL‐10 in brain tissue supernatants as described by the manufacturer. All data were normalized to the total protein concentration, which was measured by a bicinchoninic acid protein assay kit (E‐BC‐K318‐M, Elabscience, China).

### 2.12 Redox Homeostasis Assay

Reactive oxygen species (ROS) level in the brain was measured using a ROS assay kit (E‐BC‐K138‐F, Elabscience, China). Briefly, a single‐cell suspension of the brain was prepared and incubated with manufacturer‐provided dichlorofluorescin diacetate probe at a concentration of 10 μM at 37°C for 60 min. Subsequently, fluorescence intensity was measured using a microplate reader at 500 nm excitation/525 nm emission.

Malondialdehyde (MDA) level was determined according to the protocol of a commercial assay kit (E‐BC‐F007, Elabscience, China). SOD activity assay was evaluated in accordance with the protocol of a commercial assay kit (E‐BC‐K020‐M, Elabscience, China). All data were normalized to the total protein concentration.

### 2.13 NO Assay

NO level in the supernatants of meningeal homogenates was measured using a commercial NO assay kit (E‐BC‐K135‐M, Elabscience, China) as described by the manufacturer.

For NO assay in human lymphatic endothelial cells (LECs), a commercial DAF‐FM DA fluorescence probe kit (S0019S, Beyotime, China) was used. Specifically, the human LECs were cultured in ECM medium (1001, ScienCell, USA) in T25 flasks until reaching 80% confluence. The LECs were then detached using 0.05% trypsin (25200056, Gibco, USA) and seeded into 35 mm culture dishes (430165, Corning, USA) at a density of 1.5 × 10^5^ cells per dish. After culturing for 72 h to achieve 100% confluence, the LECs were used for subsequent tests. A 1 mL solution of DAF‐FM DA (5 μmol/L) was added to the dishes. The dishes were then immediately transferred to a dark incubator at 37°C with 5% CO_2_ for 20 min. This step was followed by PBS washing and a 17‐min PBM treatment. Subsequently, a confocal laser scanning microscope (LSM 710, Zeiss, Germany) was used to take images, and a microplate reader assay (495 nm excitation/515 nm emission) was employed to measure the NO levels in collected LECs.

### 2.14 Histological Analysis

Hematoxylin and eosin staining, as well as Nissl staining, were conducted to evaluate the safety of PBM. Briefly, the mice were sacrificed, and their brains were carefully extracted. The brain tissues were then fixed in 4% paraformaldehyde for 24 h, followed by paraffin embedding. Subsequently, the brains were sliced into 4‐μm‐thick sections and stained with either hematoxylin and eosin or cresyl violet. The stained sections were then imaged using a microscope (Ni‐E, Nikon, Japan).

### 2.15 Western Blot Analysis

Western blot analysis was performed to measure the activity of caspase‐3. Briefly, the total protein of each brain sample was measured by the correction in bicinchoninic acid protein assay kit (E‐BC‐K318‐M, Elabscience, China). The protein from each sample was separated by 4%–12% NuPAGE (180‐8018H, Tanon, China). After transferring the proteins to a polyvinylidene fluoride membrane (88518, Thermo Scientific, USA), the membrane was blocked with 5% skim milk for 1.5 h at room temperature and then incubated with the primary antibody: anticaspase 3 (1:1000; 19677‐1‐AP, Proteintech, China) at room temperature overnight. Afterwards, the membrane was incubated with HRP‐conjugated secondary antibody (20536‐1‐AP, Proteintech, China) at room temperature for 1 h and then treated with enhanced chemiluminescent reagent kit (PK10003, Proteintech, China). Ultimately, the bands were scanned and digitalized. The density of each band was quantified using the open FIJI software (v2.15.1) and normalized to the values of β‐actin.

### 2.16 Image Analysis

For analysis of the diameter of MLVs, custom‐written and open‐source codes implemented in Matlab were utilized (Liu et al. 2023). For analysis of the morphology of glial cells, Imaris (v7.6, Bitplane AG, Switzerland) software was used to render microglia and measure their soma volume, soma sphericity, and convex hull volume, as well as the volume of astrocytes. The smoothing was set at 0.3 μm for all images, and an appropriate threshold was applied to remove the background noise and render the cells well. Neuron2‐APP2 plugin with appropriate parameters in Vaa3D software (v3.601) was used to measure the branch length, branch diameter, and number of bifurcations of microglia (Peng et al. 2014; Xiao and Peng 2013). To quantify the coverage area of AGEs, FITCD, Lyve‐1, the number of TUNEL‐positive cells, the average fluorescence intensity of EBD, myelin basic protein (MBP), FITCD, DAF‐FM DA, and AGEs within the specified regions, the open‐source FIJI software (v2.15.1) was used. The CBF value in the entire field of view and diameter of blood vessels in 5 ROIs were measured by FIJI software.

### 2.17 Statistical Analysis

All data were presented as mean ± standard deviation. The statistical analysis of the data was conducted in GraphPad Prism 9. Shapiro–Wilk test and Brown–Forsythe test were applied to assess normality and homogeneity of variances, respectively. Normally distributed data with equal variances were analyzed using one‐way ANOVA test or *t*‐test. Otherwise, Kruskal–Wallis test or Mann–Whitney *U* test were used. All *p* values < 0.05 were considered statistically significant.

## 3 Results

### 3.1 PBM Improves Lymphatic Removal of AGEs and Brain Drainage

**FIGURE 1:**
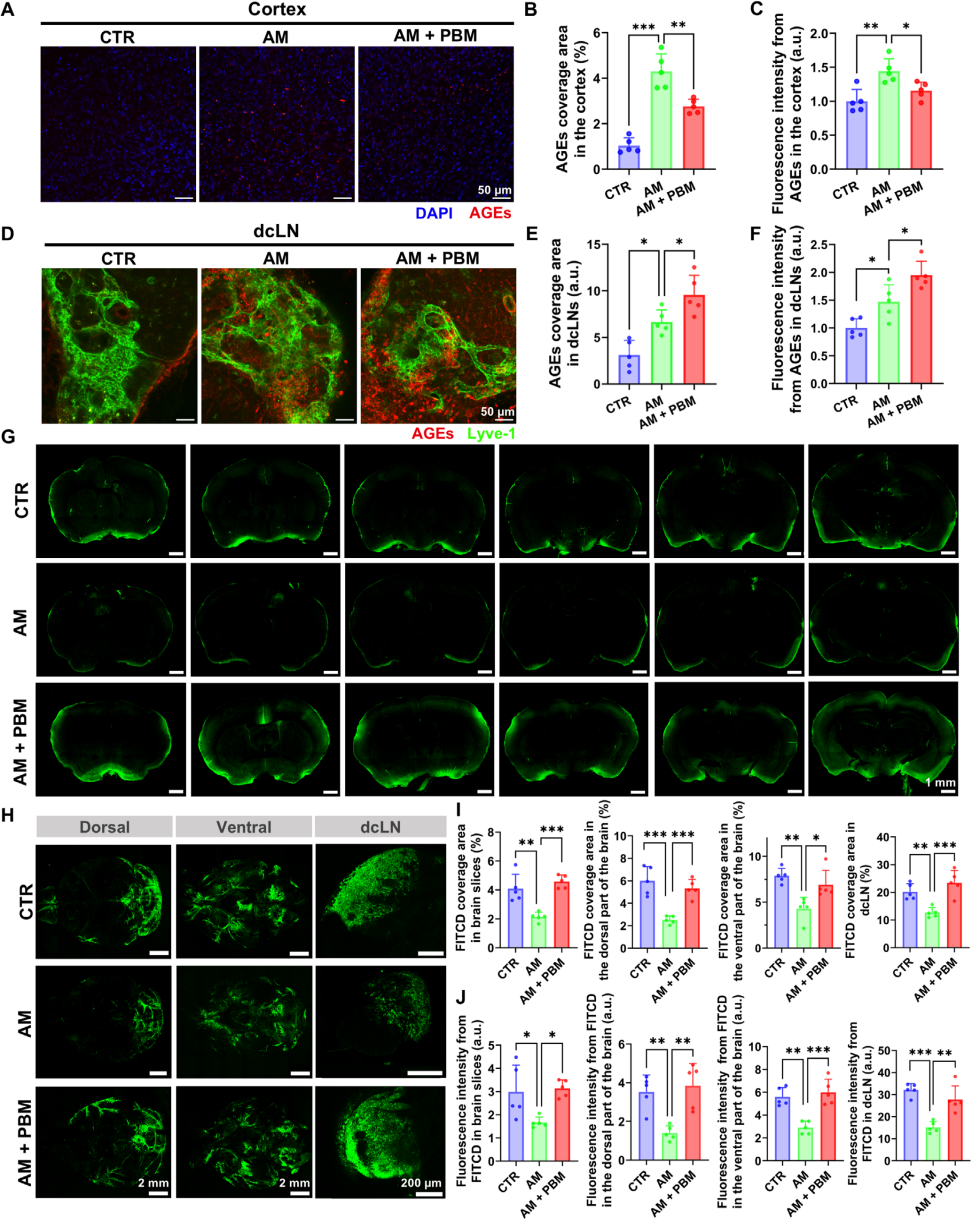
PBM improves brain drainage and lymphatic removal of AGEs from the brain to dcLNs in AM mice. (A) Representative images of the presence of AGEs in the cortex in the groups of CTR, AM, and AM+PBM. Scale bar: 50 μm. (B and C) Quantitative analysis of coverage area (B) and the intensity of the fluorescent signal (C) from AGEs in the cortex among the three tested groups. (D) Representative images of the presence of AGEs in dcLN among the three tested groups. Scale bar: 50 μm. (E and F) Quantitative analysis of coverage area (E) and the intensity of the fluorescent signal (F) from AGEs in dcLN among the three tested groups. (G) Representative images of FITCD influx into brain parenchyma among the three tested groups, scale bar: 1 mm. (H) Representative images of FITCD distribution in the dorsal and ventral parts of the brain as well as in dcLN among three tested groups, scale bar: 2 mm (brain); 200 μm (dcLN). (I and J) Quantitative analysis of FITCD coverage area (I) and the intensity of the fluorescent signal (J) from FITCD in the brain slices, dorsal and ventral parts of the brain, as well as in dcLN among the three tested groups. The data in B, C, E, F, I, and J are presented as mean ± standard deviation; *n* = 5 mice in each group; *p < 0.05, **p < 0.01, ***p < 0.001.

First, we investigated the changes of brain drainage and lymphatic clearance of AGEs in D‐gal‐induced AM mice. The results demonstrate that both the AGEs coverage area as well as the intensity of the fluorescent signal from AGEs in the cortex were higher in the AM group versus CTR (the AGEs coverage area: *p* < 0.0001; the intensity of the fluorescent signal: *p* = 0.0025) (Figure 1A–C). A similar high accumulation of AGEs in dcLNs was observed in the AM group versus CTR, suggesting that D‐gal affects not only MLVs but also peripheral lymphatics (the AGEs coverage area: *p* = 0.0155; the intensity of the fluorescent signal: *p* = 0.0273) (Figure 1D–F). Additionally, the results demonstrate a significant decrease in the influx of FITCD into the brain parenchyma in the AM group versus CTR (the FITCD coverage area: *p* = 0.0012; the intensity of the fluorescent signal: *p* = 0.0312) (Figure 1G,I,J). As demonstrated in Figure 1H–J, a distribution of FITCD in the dorsal and ventral parts of the brain as well as removal of tracer to dcLNs were also suppressed in the AM group versus CTR (for FITCD coverage area: *p* = 0.0001, *p* = 0.0019, *p* = 0.0079 for the dorsal and ventral parts of the brain and dcLN, respectively; for the intensity of the fluorescent signal: *p* = 0.0054, *p* = 0.001, and *p* < 0.0001 for the dorsal and ventral parts of the brain and dcLN, respectively). These findings show reduced brain drainage in AM mice. The changes observed in D‐gal‐induced AM mice closely resemble those seen in naturally aging mice (Da Mesquita et al. 2018; Wang, Yan, et al. 2024).

Then, we studied the possibility of ameliorating aging‐associated brain drainage deterioration and enhancing AGEs clearance using PBM. We first determined the dose of PBM on the AM mice. It is well known that near‐infrared PBM can induce heating effects due to water‐mediated light absorption (Yun and Kwok 2017). The brain functions are sensitive to temperature changes if it changes above 0.5°C that may cause alterations in the cellular processes (Li, Lin, et al. 2023). Therefore, we measured the temperature on the surface of the cortex for four laser power densities: 5, 10, 20, and 40 mW/cm^2^ (corresponding energy density: 15, 30, 60, and 120 J/cm^2^), and we analyzed histological changes in brain tissues after the 14‐day PBM course with the indicated PBM doses in healthy mice. Our results revealed no histological changes in brain tissues after the PBM course at the four different doses described above (Figure S2A). The temperature increased slightly on the brain surface during PBM 5 mW/cm^2^ (15 J/cm^2^; +0.10°C) and 10 mW/cm^2^ (30 J/cm^2^; +0.39°C), but only after PBM 20 mW/cm^2^ (60 J/cm^2^; +0.57°C) and 40 mW/cm^2^ (120 J/cm^2^; +1.16°C) did the temperature exceed the recommended values by 0.5°C (Figure S2B). Given the absence of histological changes in brain tissues and the preference to use PBM with temperature effects on the brain surface not exceeding 0.5°C, the dose of PBM 30 J/cm^2^ was chosen as the maximum for further research.

Our results clearly demonstrate that the PBM course caused a reduction of AGEs content in the cortex (the AGEs coverage area: *p* = 0.0013; the intensity of the fluorescent signal: *p* = 0.0386) that was accompanied by more extensive accumulation of toxin in dcLNs in the AM+PBM group compared with the AM group (the AGEs coverage area: *p* = 0.0453; the intensity of the fluorescent signal: *p* = 0.0255) (Figure 1A–F). The single PBM application also effectively improved brain drainage in the AM group, which manifested in an increase in the influx of FITCD, the distribution of this tracer in the dorsal and ventral parts of the brain, and the lymphatic removal of FITCD to dcLNs compared with the AM group (Figure 1G,H). The quantitative analysis of indicated parameters of brain drainage is presented in Figure 1I,J (for FITCD coverage *p* = 0.0002, *p* = 0.0009, *p* = 0.0377, and *p* = 0.0005 for the brain slices, the dorsal and ventral parts of the brain, and dcLN, respectively; for the intensity of the fluorescent signal of FITCD *p* = 0.0174, *p* = 0.002, *p* = 0.0003, and *p* = 0.0013 for the brain slices, dorsal and ventral parts of the brain, and dcLN, respectively).

These results clearly show that AM mice exhibit the accumulation of AGEs in the brain and impairment of brain drainage. 1275‐nm PBM at 30 J/cm^2^ helps to restore brain drainage efficiency and promotes lymphatic excretion of AGEs without causing any noticeable heat damage.

### 3.2 Role of NO in the Mechanisms of PBM Improvement of MLVs Drainage

To reveal how PBM improves brain drainage and lymphatic excretion of AGEs, two hypotheses were tested for this purpose: (1) through lymphangiogenesis, which should lead to the restoration of the MLVs network; (2) by increasing the drainage properties of MLVs, in which NO plays an important role.

**FIGURE 2:**
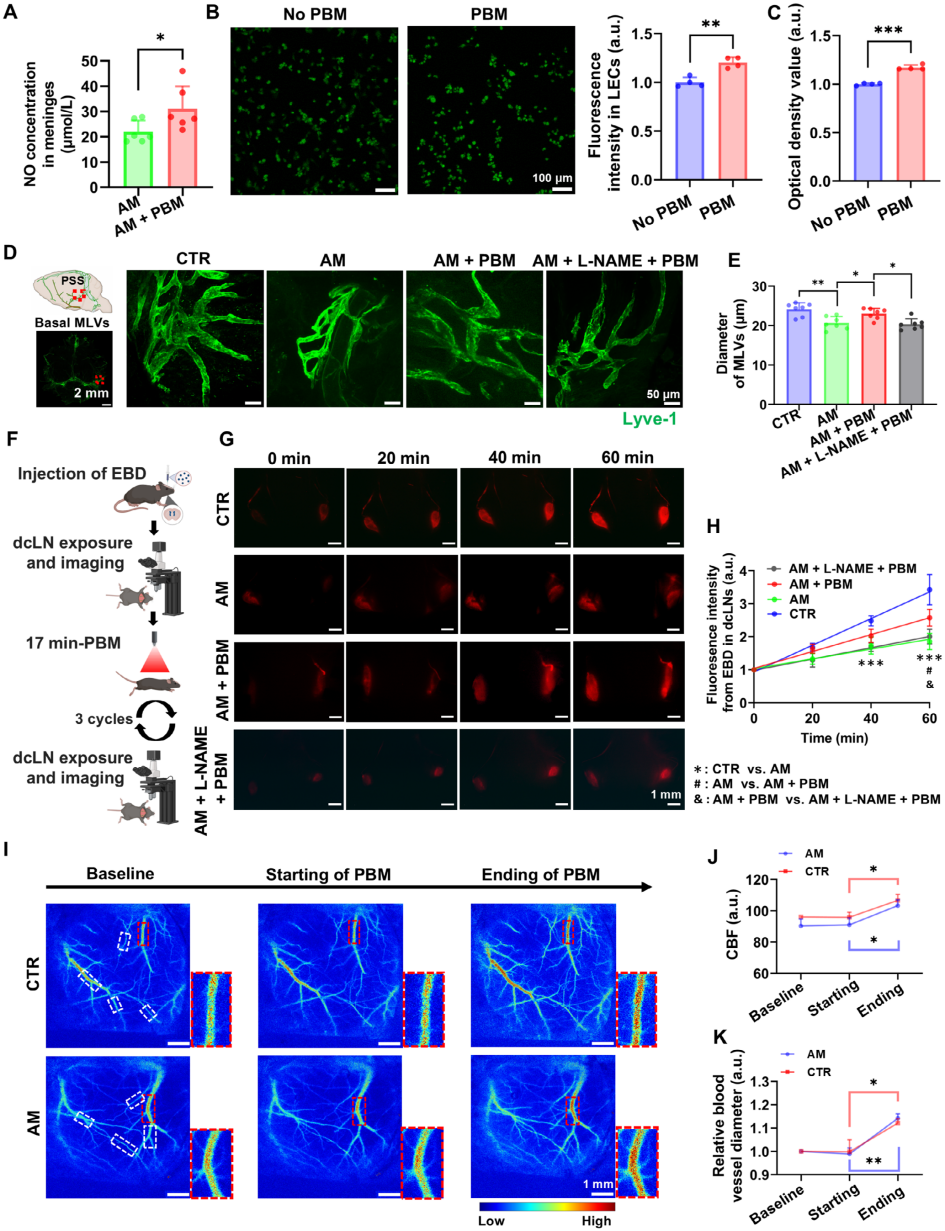
PBM modulates the tone of MLVs and increases CBF in AM mice. (A) Quantitative analysis of the NO levels in the meninges in AM and AM+PBM groups. (B) Representative images and quantitative analysis of fluorescence intensity of DAF‐FM DA in the human LECs among the No PBM and the PBM groups. (C) Optical density value of collected LECs measured by microplate reader among these two groups. (D) Representative fluorescent images of MLVs in the groups of CTR, AM, AM+PBM, AM+L‐NAME+PBM. Scale bar: 50 μm. At least two fields of view from each meningeal sample were used for statistical analysis. (E) Quantitative analysis of average diameter of MLVs among these four groups. (F) Experimental design for the in vivo monitoring of lymphatic excretion of EBD without and after a single PBM application. (G) Representative images of EBD clearance from the right lateral ventricle into dcLNs in the four tested groups, scale bar: 1 mm. (H) Quantitative analysis of the intensity of the fluorescent signal from EBD in dcLNs in the four tested groups. (I) Representative laser speckle contrast images of blood flow in groups of CTR and AM before and after a single application of PBM. Scale bar: 1 mm. (J, K) Quantitative analysis of CBF in the entire field of view (J) and the relative diameter (K) in 5 ROIs in these two groups. Data in A–C, E, H, J, and K are presented as mean ± standard deviation. *n* = 6 mice for A, *n* = 4 cell culture wells for B and C, *n* = 7 mice for E, *n* = 5 mice for H, *n* = 3 mice for J and K. *p < 0.05, **p < 0.01, ***p < 0.001, #*p* < 0.05, & p < 0.05.

Our results revealed that the coverage of basal MLVs around petrosquamosal sinus (PSS), which is involved in brain drainage, was 1.27 times less in the AM group compared with the CTR group (*p* = 0.0239) (Figure S3). However, PBM course did not significantly increase the MLVs coverage around PSS in the AM+PBM group vs. the AM group (*p* = 0.7392) (Figure S3). Nonetheless, we found that a single PBM application significantly increased the NO concentration (nitrates and nitrites) in the supernatant of meningeal homogenates in the AM+PBM group compared to the AM group (*p* = 0.0467) (Figure 2A). Results from in vitro experiments further confirmed the effect of PBM on the NO release from the human LECs (Figure 2B,C). We found that the fluorescence intensity of NO probe in LECs was significantly increased after 17‐min PBM treatment, indicating an increase in the NO level (Fluorescence intensity: *p* = 0.0016; optical density value: *p* < 0.0001). The above results indicate that PBM promotes NO release in MLVs.

Additionally, we examined the changes in diameter of MLVs following a single PBM treatment. Our results demonstrate that PBM increased the diameter of narrowed MLVs in the AM group. However, such an effect was inhibited by NO blockage using L‐NAME (AM vs. AM+PBM: *p* = 0.0358; AM vs. AM+L‐NAME+PBM: *p* = 0.9789, ns; AM+PBM vs. AM+PBM + L‐NAME: *p* = 0.0147) (Figure 2D,E). To assess the effect of NO release on PBM‐mediated enhancement of brain drainage, we injected EBD intracerebroventricularly and monitored its accumulation in the dcLNs in vivo after L‐NAME administration (Figure 2F–H). The results revealed that L‐NAME administration inhibited the PBM‐mediated enhancement of lymphatic clearance efficacy (60 min; AM vs. AM+PBM: *p* = 0.0103; AM vs. AM+L‐NAME+PBM: *p* = 0.8767, ns; AM+PBM vs. AM+L‐NAME+PBM: *p* = 0.0454). The above results suggest that PBM‐induced NO release in LECs contributes to the dilation of MLVs and enhancement of meningeal lymphatic clearance.

It is known that the effects of PBM on the NO production are closely related to changes in microcirculation. Indeed, our results show that the single PBM increased the CBF in healthy and AM mice (Starting vs. Ending; CTR: *p* = 0.0182; AM: *p* = 0.0211), accompanied by an increase in blood vessel diameter (Starting vs. Ending; CTR: *p* = 0.0176; AM: *p* = 0.0013) (Figure 2I–K). Note that there was no statistically significant difference in CBF between CTR and AM mice (CTR vs. AM: *p* = 0.0961, ns).

### 3.3 PBM Promotes Neuroprotection and Modulates Redox Homeostasis in AM Mice

Redox imbalance plays an important role in aging‐related neurodegeneration and cognitive decline. This abnormality may result from impaired brain drainage and could serve as a target for the therapeutic effects of PBM (Kandlur et al. 2020).

**FIGURE 3:**
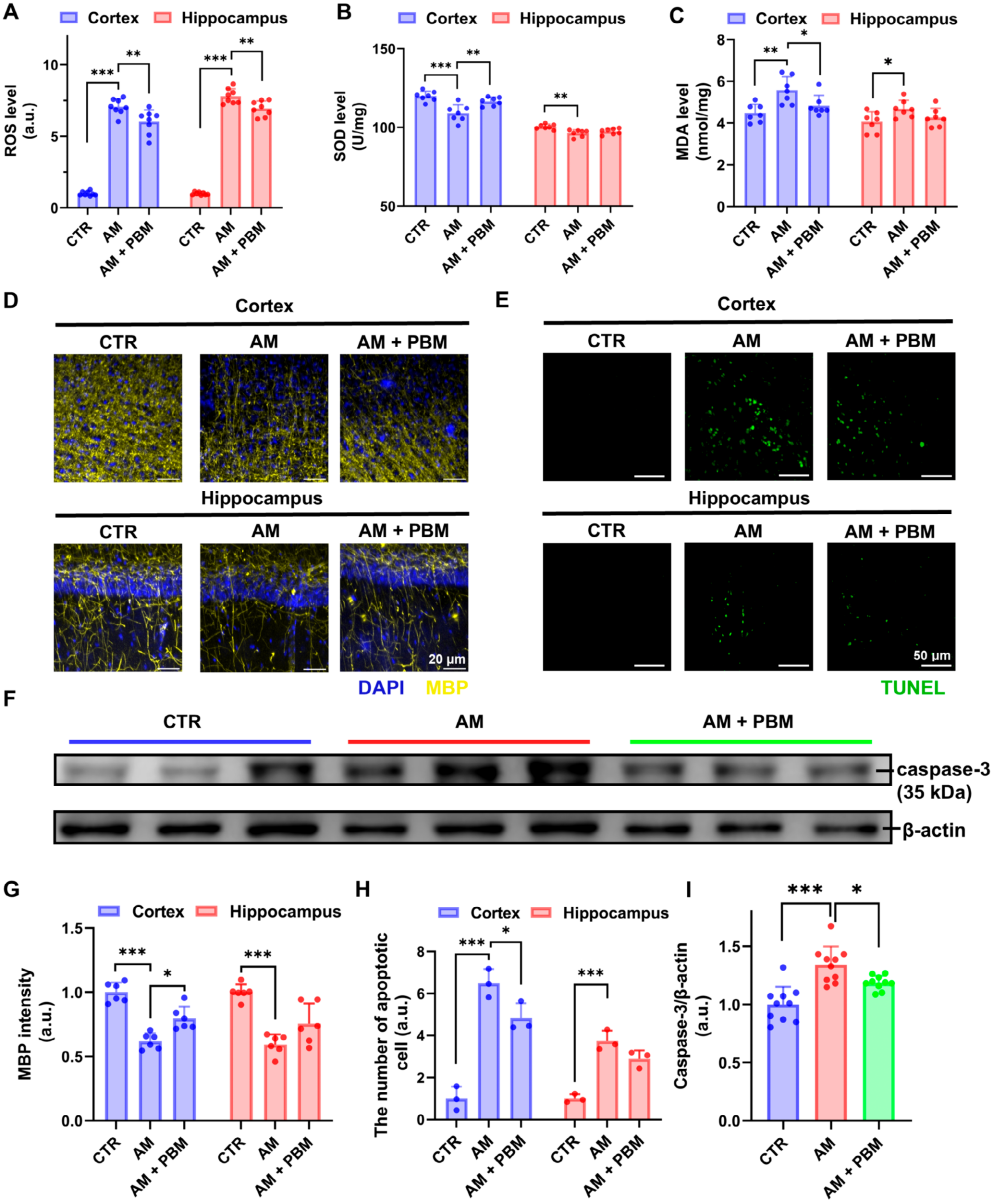
PBM course modulates redox homeostasis and mitigates neuronal damage in AM mice. (A‐C) Quantification of ROS (A), SOD (B), and MDA (C) levels in the brain in CTR, AM, and AM+PBM groups. (D) Representative images of MBP staining in the brain in three tested groups. Scale bar: 20 μm. (E) Representative images of TUNEL staining in the brain in three tested groups. Scale bar: 50 μm. (F) Representative images of caspase‐3 western blot in the brain in three tested groups. (G–I) Quantitative analysis of MBP intensity (G), the number of TUNEL‐positive cells (H), and caspase‐3 levels (I) in the brain among three tested groups. Data in A–C and G–I are presented as mean ± standard deviation, *n* = 7–8 mice for A–C, *n* = 6 mice for G, *n* = 3 mice for H, *n* = 10 mice for I. *p < 0.05, **p < 0.01, ***p < 0.001.

Our results show elevated ROS (Cortex: *p* < 0.0001; hippocampus: *p* < 0.0001) and MDA (Cortex: *p* = 0.0031; hippocampus: *p* = 0.0451) levels in AM mice, as well as decreased SOD (Cortex: *p* = 0.0002; hippocampus: *p* = 0.0021) levels in both the cortex and hippocampus, indicating increased oxidative damage and deteriorated antioxidant capacity, compared to the mice in the CTR group (Figure 3A–C). Remarkably, the above abnormalities were improved in the PBM‐treated mice (Figure 3A–C). Specifically, the PBM course significantly decreased ROS in both the cortex and the hippocampus (Cortex: *p* = 0.0045; hippocampus: *p* = 0.0020). For MDA and SOD, PBM had effects in the cortex (reduced the MDA level and alleviated the decrease of SOD), while the changes in the hippocampus were statistically insignificant (MDA: Cortex: *p* = 0.0442; hippocampus: *p* = 0.2288, ns; SOD: cortex: *p* = 0.0056; hippocampus: *p* = 0.5711, ns).

The changes in neurons in AM mice, as well as those treated with PBM, were evaluated (Figure 3D–F). Quantitative analysis demonstrates that the AM mice evidenced a significant decrease in the MBP intensity in both the cortex and hippocampus compared to CTR (Cortex: *p* < 0.0001; hippocampus: *p* < 0.0001). However, the PBM course significantly reversed this abnormality in the cortex, but not in the hippocampus (Cortex: *p* = 0.0035; hippocampus: *p* = 0.0508, ns) (Figure 3G). Furthermore, AM mice demonstrated a marked increase in TUNEL‐positive cells in both the cortex and hippocampus (Cortex: *p* < 0.0001; hippocampus: *p* = 0.0003), while it was significantly decreased after the PBM course in the cortex but not in the hippocampus (Cortex: *p* = 0.0479; hippocampus: *p* = 0.0788, ns) (Figure 3H). Besides, AM mice showed a significant increase in the caspase‐3 levels in the cortex (*p* < 0.0001) (Figure 3I). The PBM course significantly reduced the caspase‐3 expression in the cortex (*p* = 0.0334). The above results clearly demonstrate the antiapoptotic properties of PBM in AM mice.

These results demonstrate that the AM mice exhibit oxidative stress leading to neuronal damage and apoptosis, which can be ameliorated by the PBM course.

### 3.4 PBM Suppresses Neuroinflammation in AM Mice

Aside from redox imbalance, neuroinflammation is also a critical risk factor for aging‐related neuronal damage and the development of neurodegenerative diseases, and is a target of PBM. Besides, enhanced meningeal lymphatic drainage has been shown to contribute to the amelioration of neuroinflammation (Dong et al. 2024; Gao et al. 2024). Therefore, we analyzed the effects of the PBM course on the microglial phenotypic changes reflecting neuroinflammation.

**FIGURE 4:**
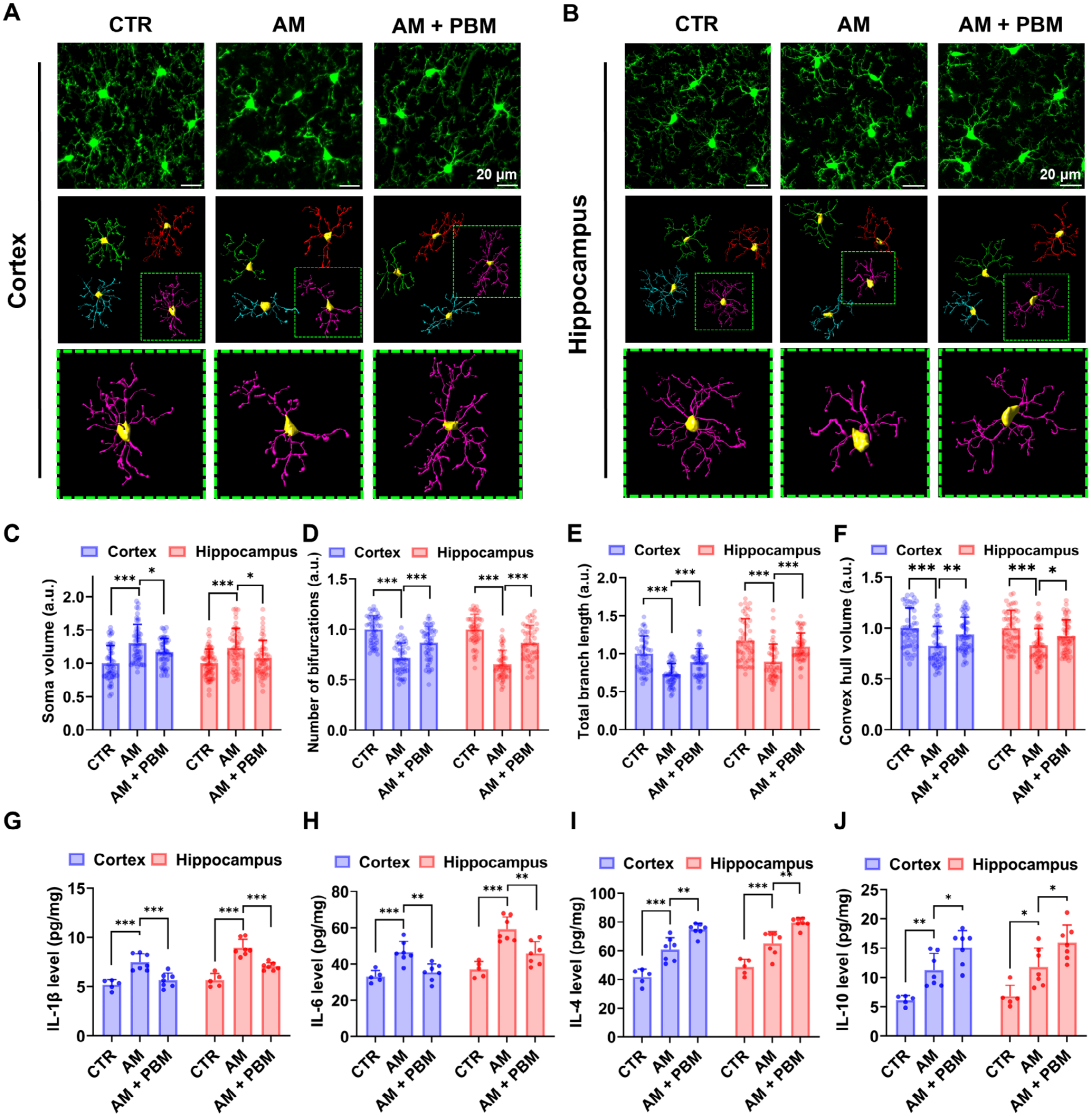
PBM course mitigates neuroinflammation in the brain of AM mice. (A and B) Representative maximum intensity projection images and 3D reconstructions (with detailed views) of cortical (A) and hippocampal (B) microglia in the CTR, AM, and AM+PBM groups. Scale bar: 20 μm. (C–F) Quantitative analysis of microglial soma volume (C), number of bifurcations (D), total branch length (E), and convex hull volume (F) in the brain among three tested groups. (G–J) Quantitative analysis of cytokines IL‐1β (G), IL‐6 (H), IL‐4 (I), and IL‐10 (J) levels in the brain among three tested groups. Data in C–J are presented as mean ± standard deviation. *n* = 50 cells from five mice per group for C–F, *n* = 5–7 mice per group for G–J. *p < 0.05, **p < 0.01, ***p < 0.001.

Our results show that the microglia in the cortex and hippocampus in AM mice were activated, which was manifested in a significant decrease in microglial branch length (Cortex: *p* < 0.0001; hippocampus: *p* < 0.0001), number of bifurcations (Cortex: *p* < 0.0001; hippocampus: *p* < 0.0001), soma sphericity (Cortex: *p* = 0.0036; hippocampus: *p* < 0.0001), and convex hull volume (Cortex: *p* < 0.0001; hippocampus: *p* < 0.0001), as well as an increase in branch diameter (Cortex: *p* < 0.0001; hippocampus: *p* < 0.0001) and soma volume (Cortex: *p* < 0.0001; hippocampus: *p* < 0.0001) (Figure 4A–F; Figure S4). However, the course of PBM significantly reduced the reactivity of microglia (Figure 4A–F; Figure S4). Indeed, branch length (Cortex: *p* = 0.0001; hippocampus: *p* = 0.0001), number of bifurcations (Cortex: *p* < 0.0001; hippocampus: *p* < 0.0001), convex hull volume (Cortex: *p* = 0.0073; hippocampus: *p* = 0.0164), and soma sphericity (Cortex: *p* = 0.0038; hippocampus: *p* = 0.0459) were increased after the PBM course in AM mice compared to mice without PBM. After PBM, microglia in AM mice also demonstrated reduced branch diameter (Cortex: *p* < 0.0001; hippocampus: *p* < 0.0001) and soma volume (Cortex: *p* = 0.0265; hippocampus: *p* = 0.0108) compared to untreated AM mice. Besides, the volume of astrocytes was significantly increased in the AM mice compared with the mice in the CTR group, while the PBM course remarkably alleviated such hypertrophy of astrocytes (Cortex: *p* = 0.0415; hippocampus: *p* = 0.0288) (Figure S5).

Additionally, we measured the inflammatory cytokine levels in the brain to further explore the effects of the PBM course on neuroinflammation. We observed that the levels of proinflammatory cytokines IL‐1β and IL‐6 were increased in the AM mice vs. the CTR group (for IL‐1β *p* = 0.0002 and *p* < 0.0001; for IL‐6 *p* = 0.0007 and *p* < 0.0001 in the cortex and hippocampus, respectively), while their levels were reduced after the PBM course (for IL‐1β *p* = 0.0006 and *p* = 0.0002; for IL‐6 *p* = 0.0016 and *p* = 0.0027 in the cortex and hippocampus, respectively) (Figure 4G,H). The levels of the anti‐inflammatory cytokines IL‐4 and IL‐10 were naturally increased in AM mice as a response to inflammation (for IL‐4 *p* = 0.0002 and *p* = 0.0008; for IL‐10 *p* = 0.0077 and *p* = 0.0241 in the cortex and hippocampus, respectively). The PBM course further increased the level of these tested cytokines (for IL‐4 *p* = 0.0019 and *p* = 0.0012; for IL‐10 *p* = 0.0286 and *p* = 0.0391 in the cortex and hippocampus, respectively) (Figure 4I,J).

Thus, these results clearly demonstrate that PBM significantly mitigates neuroinflammation in the brain of AM mice, contributing to the reduction of proinflammatory and extra activation of anti‐inflammatory cytokines.

### 3.5 PBM Reduces Cognitive Dysfunction in AM Mice

Aging‐related cognitive dysfunction is a manifestation of oxidative stress and inflammatory processes in the brain. To evaluate the therapeutic potential of the PBM course on cognitive decline in AM mice, we conducted the MWM and NOR tests.

**FIGURE 5:**
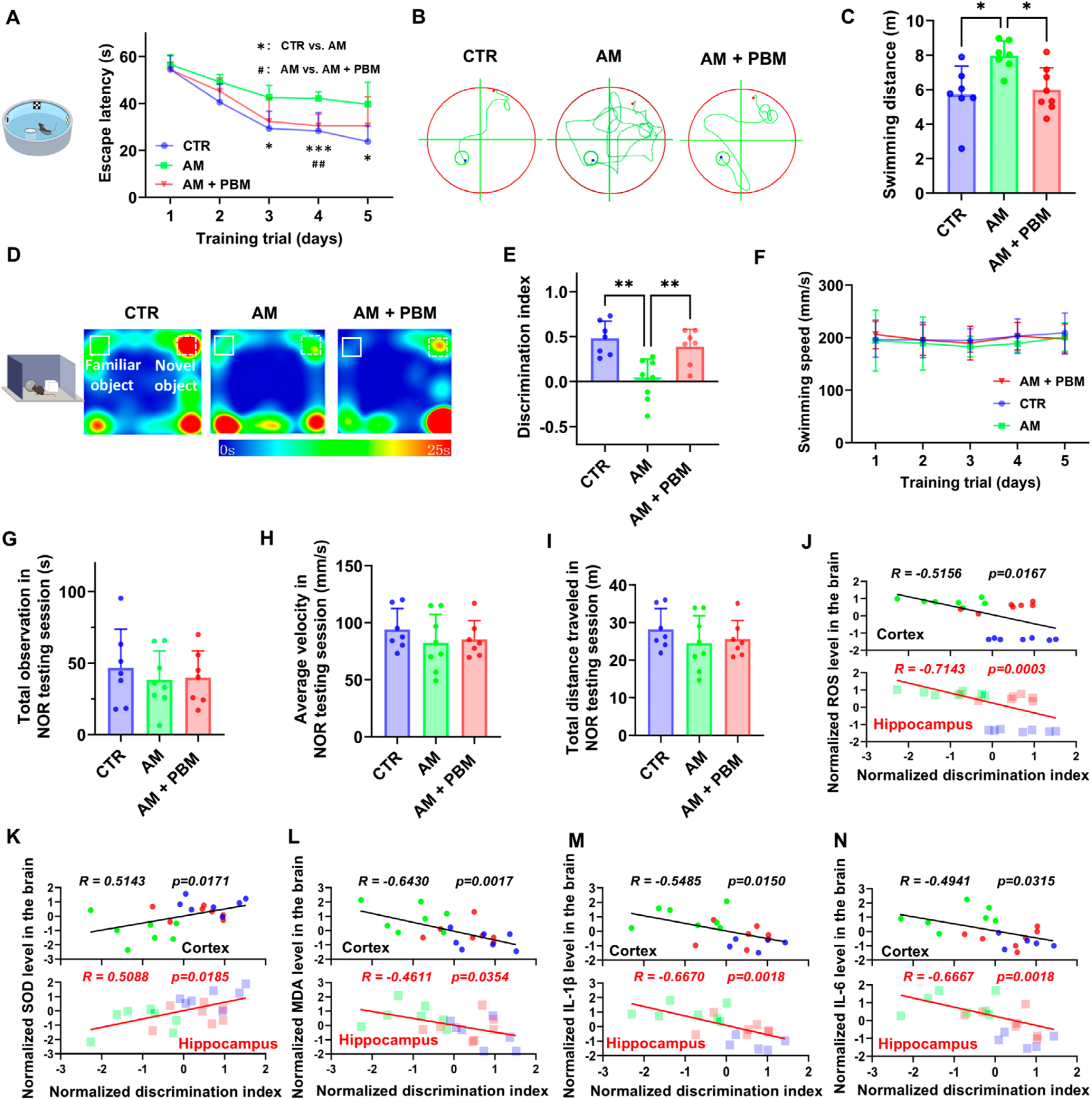
PBM course‐mediated improvements in cognitive function associated with oxidative stress and neuroinflammation in AM mice. (A–C) MWM test in the CTR, AM, and AM+PBM groups, including escape latency (A), typical swimming path on Day 4 (B) and quantitative analysis of swimming distance on Day 4 (C) during MWM training session. (D) Typical movement paths in NOR testing session among three tested groups. (E) Quantitative analysis of discrimination index in NOR testing session among three tested groups. (F) Quantitative analysis of swimming speed during MWM training session. (G–I) Quantitative analysis of total observation time (G), average velocity (H) and total distance traveled (I) in NOR testing session. (J–N) Spearman correlation coefficient analysis of discrimination index in NOR test with ROS (J), SOD (K), MDA (L), IL‐1β (M), and IL‐6 (N) levels in brain. Blue, green, and red symbols represent the CTR, AM, and AM+PBM groups; circles and squares indicate cortical and hippocampal data, respectively. Data in A, C, E–I are presented as mean ± standard deviation, *n* = 7–8 mice per group. *p < 0.05, **p < 0.01, ***p < 0.001, ##*p* < 0.01.

Our results show that the escape latency in all tested groups progressively declined during the training session (Figure 5A). However, AM mice displayed longer escape latency on Days 3, 4, and 5 compared with the CTR group (Day 3: *p* = 0.0131; Day 4: *p* = 0.0004; Day 5: *p* = 0.019). The PBM course ameliorated this abnormality on Day 4, indicating improved learning ability (*p* = 0.0016) (Figure 5A). Furthermore, the AM mice that received the PBM course traveled shorter swimming paths on Day 4 than untreated mice (*p* = 0.0212) (Figure 5B,C).

The NOR testing session revealed that AM mice compared with the CTR group showed a decline in discrimination index (AM vs. CTR: *p* = 0.001), suggesting an impairment of the short‐term recognition memory (Figure 5D,E). However, the PBM course significantly improved discrimination index in AM mice (*p* = 0.0072). Our results demonstrate that there were no significant differences in average swimming speed during the MWM training session, nor in total observation time, average velocity, or total distance traveled during the NOR training and testing sessions (Figure 5F–I; Figure S6A–C). These findings indicate that the cognitive improvements were not due to general differences in motor activities. The results during the testing sessions of the MWM test and the training sessions of the NOR test showed no differences among all the tested groups (Figure S6D,E).

Additionally, the Spearman correlation coefficient analysis revealed strong correlations (*R* > 0.6) between the levels of MDA in the cortex, ROS in the hippocampus, and the proinflammatory cytokines IL‐1β and IL‐6 in the hippocampus with the DI in the NOR test (Figure 5J–N), indicating a potential association between improvements in neuroinflammation and oxidative stress and enhancements in cognitive function.

Thus, these findings demonstrate that PBM improves the spatial learning ability and short‐term recognition memory in AM mice.

## 4 Discussion

In this study, we demonstrate that the D‐gal‐induced aging mouse model was characterized by impaired brain drainage and accumulation of metabolite AGEs in the brain. These abnormalities can be improved by PBM (1275 nm, 30 J/cm^2^) via the stimulation of NO release in LECs, which mediates the dilation of the MLVs. PBM also alleviated neuroinflammation, redox imbalance, and neuronal damage in AM, thereby contributing to the improvements in cognitive function.

Aging and aging‐related neurodegenerative diseases are commonly associated with impaired brain clearance function and accumulation of toxic metabolites. In the aging brain, the metabolic imbalance of AGEs is an important factor contributing to neuroinflammation, oxidative stress, and may increase the risk of neurodegenerative diseases, such as AD and PD (Chaudhuri et al. 2018). It was reported that certain AGEs have a molecular weight ranging from 1 to 12 kDa and are water soluble (Goldin et al. 2006; Twarda‐Clapa et al. 2022), making them detectable in the cerebrospinal fluid (CSF) (Ramírez‐Boo et al. 2012). This suggests that AGEs could be cleared by the brain lymphatic pathway. MLVs are “tunnels” that facilitate the elimination of toxins and metabolites, including Aβ in AD subjects and even erythrocytes following subarachnoid hemorrhage (Dupont et al. 2020; Chen et al. 2020; Semyachkina‐Glushkovskaya, Alexander, et al. 2023). In this study, we have shown the lymphatic route for the elimination of AGEs and its suppression in AM mice, which is important in understanding the AGEs‐mediated mechanisms of the development of aging‐related neurodegenerative diseases as well as for the development of effective therapeutics for the improvement of lymphatic clearance of AGEs in high‐risk groups.

Modulation of the MLV function through pharmacological methods has been proposed (Da Mesquita et al. 2021; Hu et al. 2020; Liao et al. 2023; Song et al. 2020). However, systemic drug administration via the bloodstream often fails to effectively target or penetrate MLVs, necessitating alternative delivery routes such as intracisternal injection. One of the most promising and widely used methods in this direction is the stimulation of lymphangiogenesis by introducing the vascular endothelial growth factor C into the cisterna magna (Da Mesquita et al. 2021; Hu et al. 2020; Song et al. 2020). Besides, a recent study has shown that intracisternal administration of ketoprofen and 9‐cis retinoic acid enhances the MLV function, promoting CSF drainage and alleviating brain edema in traumatic brain injury mice (Liao et al. 2023). It should be noted that these therapeutics are invasive, which restricts their widespread clinical use. They can only be performed for specific indications by highly qualified specialists. However, MLVs are ideal targets for PBM.

In this study, we demonstrate that 1275‐nm PBM enhances brain drainage and AGEs clearance in AM mice, which is mediated by NO‐induced dilation of MLVs, rather than by lymphangiogenesis. NO plays a critical role in maintaining cerebrovascular homeostasis (Kashiwagi et al. 2023). Initial studies demonstrated that PBM enhances vascular endothelial NO release, providing therapeutic benefits for cardiovascular diseases (Kashiwagi et al. 2023; Yokomizo et al. 2024). Recently, the effects of PBM‐mediated NO release on MLVs have been discovered. Our previous studies demonstrated that NO blockade abolishes PBM‐mediated MLVs dilation in diabetic mice (Liu et al. 2023). Here, we provide strong evidence that PBM directly stimulates NO production in LECs, thereby dilating MLVs and potentially ameliorating impairments in brain drainage and AGEs clearance in AM mice. Moreover, from a vascular physiology perspective, NO plays an active role in regulating the contractility of lymphatic vessels (Bohlen et al. 2011). NO can increase the permeability of MLVs, facilitating fluid influx. This opens upstream valves while closing downstream valves. Upon filling, reduced shear stress degrades NO, initiating Ca^2+^‐dependent MLVs contraction. This peristaltic mechanism underpins MLVs drainage and clearance functions (Bohlen et al. 2011). In the future, it would be valuable to explore the PBM‐induced MLVs contraction process using high‐resolution in vivo imaging techniques.

It is known that the effects of PBM on the NO production are closely related to changes in microcirculation. Combining noninvasive skull optical clearing and laser speckle contrast imaging techniques, our results show that PBM increases CBF and blood vessel diameter in AM mice. These findings are consistent with the observation reported by Yokomizo et al. (2024), who utilized laser Doppler flowmetry to demonstrate that PBM increases CBF in stroke mice. Furthermore, arterial pulsation and CBF are considered driving forces for brain drainage (Murdock et al. 2024; Sun et al. 2024). In AM mice, the PBM‐induced increase in CBF further supports the enhancement of brain drainage. Additionally, besides the dilation of MLVs, the enhancement of brain lymphatic drainage may also result from the stimulation of AQP4 activation (Murdock et al. 2024).

Aging‐related neuroinflammation and oxidative stress are closely linked to neurodegeneration and cognitive decline, which serve as important targets for slowing the development of neurodegenerative diseases. In our study, PBM improves redox homeostasis and neuronal health in the cortex. While oxidative damage in the hippocampus is not significantly reduced, likely due to limited photon absorption in this deeper brain region, PBM still effectively regulates ROS generation in the hippocampus. This may be because ROS production is highly sensitive to photon exposure, potentially due to light's interaction with mitochondrial chromophores or activation of light‐sensitive pathways (Salehpour et al. 2018). Additionally, PBM aids in the restoration of microglial health in both the cortex and hippocampus of AM mice. The results of the correlation analysis indicate that the mitigation of oxidative stress and neuroinflammation is associated with improvements in cognitive function. Our results are consistent with data demonstrating the efficacy of PBM in reducing oxidative stress and inflammatory processes when treating a wide range of brain diseases, including AD, traumatic brain injury, and depression (Chen et al. 2024; Stevens et al. 2024; Tao et al. 2021).

Recent studies have reported that pharmacological therapeutics aimed at improving MLVs effectively alleviate neuroinflammation and oxidative stress in mice (Dong et al. 2024; Gao et al. 2024; Liao et al. 2023), suggesting a potential link between improved meningeal brain drainage and mitigation of these brain abnormalities. Nonetheless, PBM can also alleviate neuroinflammation and oxidative stress through the modulation of the NF‐κB signal pathway (Li et al. 2025; Salehpour et al. 2018). Furthermore, PBM may directly excite ground‐state triplet oxygen to singlet oxygen, thereby enhancing mitochondrial function and potentially alleviating energy deficits in the aging brain (Semyachkina‐Glushkovskaya, Sokolovski, et al. 2023). Consequently, PBM‐induced mitigation of brain abnormalities may not be causally linked to the enhancement of meningeal brain drainage, and that should be explored in future studies. Additionally, the enhanced brain lymphatic drainage function may also promote the removal of Aβ and cellular and metabolic by‐products, further contributing to the improvements in cognitive function observed in mice (Li, Lin, et al. 2023; Murdock et al. 2024; Petrova and Koh 2020).

In fact, previous pilot researches have demonstrated the beneficial effects of PBM in aging treatment. Specifically, Cardoso et al. (2022) reported that 810‐nm PBM modulated the expression of cortical inflammatory markers in aging rats, while its effectiveness on hippocampal inflammatory markers was limited. Salehpour et al. (2017) found that PBM at 660 nm and 810 nm decreased ROS levels in the whole brain of an aging mouse model. Wang, Yan, et al. (2024) demonstrated that 808‐nm PBM enhanced MLVs drainage by promoting mitochondrial metabolic homeostasis in aging mice. In this study, we utilized 1275‐nm light due to its well‐known ability to penetrate deeper into the brain compared to the light at 660, 808, and 1070 nm (Genina et al. 2019; Kashiwagi et al. 2023). We focused on meningeal brain drainage and the clearance of AGEs. We also demonstrated the significant role of NO in PBM‐mediated dilation of the diameter of MLVs and brain drainage in aging treatment. Besides, we comprehensively examined the effects of PBM on aging‐related neuronal damage, neuroinflammation, and redox homeostasis in the cortex and hippocampus.

In recent years, PBM has demonstrated effectiveness in the clinical treatment of various brain diseases, such as aging (Saucedo et al. 2021), AD (Berman et al. 2017), and chronic traumatic encephalopathy (Naeser et al. 2023). Regarding clinical safety, limited reports describe transient application‐site numbness following PBM, with all cases resolving spontaneously within 24 h; no delayed adverse effects or complications have been documented in clinical trials to date (Esteves‐Pereira et al. 2025). Consequently, PBM may serve as a safe and effective intervention with high potential for rapid implementation into clinical antiaging applications.

## 5 Conclusion

This study introduces a promising near‐infrared (1275 nm) PBM approach that restores meningeal brain drainage and facilitates the lymphatic clearance of AGEs in an aging mouse model. These benefits are accompanied by effective amelioration of redox imbalance, neuroinflammation, and neuronal damage, as well as improvements in spatial learning ability and short‐term recognition memory in AM mice. Given its safety profile, efficacy, clinical accessibility, and noninvasive nature, such therapeutics represent a promising nonpharmacological strategy for addressing aging‐related neurological disorders and slowing neurodegenerative disease progression, with significant potential for rapid translation into clinical practice.

## Author Contributions

H.L. contributed to the conceptualization, experimental setup, investigation, statistical analysis, and both writing and editing of the manuscript. S.L., Q.Y., and J.L. participated in the experimental setup and statistical analysis. J.W. was involved in the writing. O.S.‐G. was involved in the conceptualization, editing, and writing. D.Z. was involved in the conceptualization, editing, supervision, and funding acquisition. D.L. and T.Y. contributed to supervision, writing, editing, conceptualization, funding acquisition, and project management.

## Conflicts of Interest

The authors declare no conflicts of interest.

## Supporting information


**Figure S1:** Experimental design. (A) The 1275‐nm laser irradiation was performed in a chamber where mice with shaved heads were allowed to move freely. (B) A daily PBM during 2 weeks was carried out in sequence: 17‐min laser irradiation followed by 5‐min pause repeated three cycles so that the total experiment time was 61 min.
**Figure S2:** Safety assessment of PBM course with different laser power densities (energy densities). (A) Representative images of hematoxylin and eosin staining, as well as Nissl staining in the cortex after PBM course with different laser power densities (energy densities). Scale bar: 20 μm. (B) Measurement of temperature change on cortical surface of mice during a single PBM session with different power densities (energy densities).
**Figure S3:** Effects of PBM course on MLVs network. Representative fluorescent images and quantitative analysis of the MLV coverage area around PSS in the groups of CTR, AM, and AM+PBM. Scale bar: 50 μm. At least two fields of view from each meningeal sample were used for the statistical analysis. The data are presented as mean ± standard deviation; *n* = 6 mice in each group; *p < 0.05.
**Figure S4:** PBM course reduces the reactivity of microglia. (A) Quantitative analysis of the microglial branch diameter among CTR, AM, and AM+PBM groups. (B) Quantitative analysis of the microglial soma sphericity among three groups. Data in (A, B) are presented as mean ± standard deviation. *n* = 50 cells from five mice per group for A, B. *p < 0.05, **p < 0.01, ***p < 0.001.
**Figure S5:** PBM course alleviates hypertrophy of astrocytes. (A) Representative images of the cortical and hippocampal astrocytes in the CTR, AM, and AM+PBM groups. Scale bar: 20 μm. (B) Quantitative analysis of the astrocytes volume among three groups. Data in (B) are presented as mean ± standard deviation. *n* = 50 cells from five mice per group for B. *p < 0.05, **p < 0.01, ***p < 0.001.
**Figure S6:** Effects of PBM course on the changes of motor activity and object preference in NOR training session and cognitive function in MWM probe trial. (A–C) Quantitative analysis of total observation time (A), average velocity (B), and total distance traveled (C) in NOR training session. (D) Quantitative analysis of object preference in NOR training session. (E) Quantitative analysis of escape latency in probe trial on Day 6 in MWM test. Data are presented as mean ± standard deviation, *n* = 7–8 mice per group.
**Table S1:** Key resources.

## Data Availability

The data are available from the corresponding author upon reasonable request.
