# Supplementary material for: 1275‐nm Photobiomodulation Alleviates Brain Drainage Impairment as a Promising Therapeutic Strategy for Aging‐Related Neurological Decline
Source: Aging Cell. 2025 Oct 15;24(12):e70261. doi: 10.1111/acel.70261 (PMC12686585; doi:10.1111/acel.70261)
Supplement: Supplementary file 1 — Figure S1: Experimental design. (A) The 1275‐nm laser irradiation was performed in a chamber where mice with shaved heads were allowed to move freely. (B) A daily PBM during 2 weeks was carried out in sequence: 17‐min laser irradiation followed by 5‐min pause repeated three cycles so that the total experiment time was 61 min. Figure S2: Safety assessment of PBM course with different laser power densities (energy densities). (A) Representative images of hematoxylin and eosin staining, as well as Nissl staining in the cortex after PBM course with different laser power densities (energy densities). Scale bar: 20 μm. (B) Measurement of temperature change on cortical surface of mice during a single PBM session with different power densities (energy densities). Figure S3: Effects of PBM course on MLVs network. Representative fluorescent images and quantitative analysis of the MLV coverage area around PSS in the groups of CTR, AM, and AM+PBM. Scale bar: 50 μm. At least two fields of view from each meningeal sample were used for the statistical analysis. The data are presented as mean ± standard deviation; n = 6 mice in each group; *p < 0.05. Figure S4: PBM course reduces the reactivity of microglia. (A) Quantitative analysis of the microglial branch diameter among CTR, AM, and AM+PBM groups. (B) Quantitative analysis of the microglial soma sphericity among three groups. Data in (A, B) are presented as mean ± standard deviation. n = 50 cells from five mice per group for A, B. *p < 0.05, **p < 0.01, ***p < 0.001. Figure S5: PBM course alleviates hypertrophy of astrocytes. (A) Representative images of the cortical and hippocampal astrocytes in the CTR, AM, and AM+PBM groups. Scale bar: 20 μm. (B) Quantitative analysis of the astrocytes volume among three groups. Data in (B) are presented as mean ± standard deviation. n = 50 cells from five mice per group for B. *p < 0.05, **p < 0.01, ***p < 0.001. Figure S6: Effects of PBM course on the changes of motor activity and object pr [file ACEL-24-e70261-s001.docx]

**1275-nm photobiomodulation alleviates brain drainage impairment as a promising therapeutic strategy for aging-related neurological decline**

Hao Lin^1^, Shaojun Liu^1^, Qihang Yang^1^, Junming Li^1^, Jue Wang^2^, Oxana Semyachkina-Glushkovskaya^3^, Dongyu Li^1,4,*^, Tingting Yu^1,*^, Dan Zhu^1^

^1^MOE Key Laboratory for Biomedical Photonics, Wuhan National Laboratory for Optoelectronics - Advanced Biomedical Imaging Facility, Huazhong University of Science and Technology, Wuhan, Hubei, China

^2^Department of Hematology, Tongji Hospital of Tongji Medical College, Huazhong University of Science and Technology, Wuhan, Hubei, China

^3^Saratov State University, Saratov, Russia

^4^School of Optical Electronic Information, Huazhong University of Science and Technology, Wuhan, Hubei, China

Correspondence:

Dongyu Li (li_dongyu@hust.edu.cn)

Tingting Yu (yutingting@hust.edu.cn)


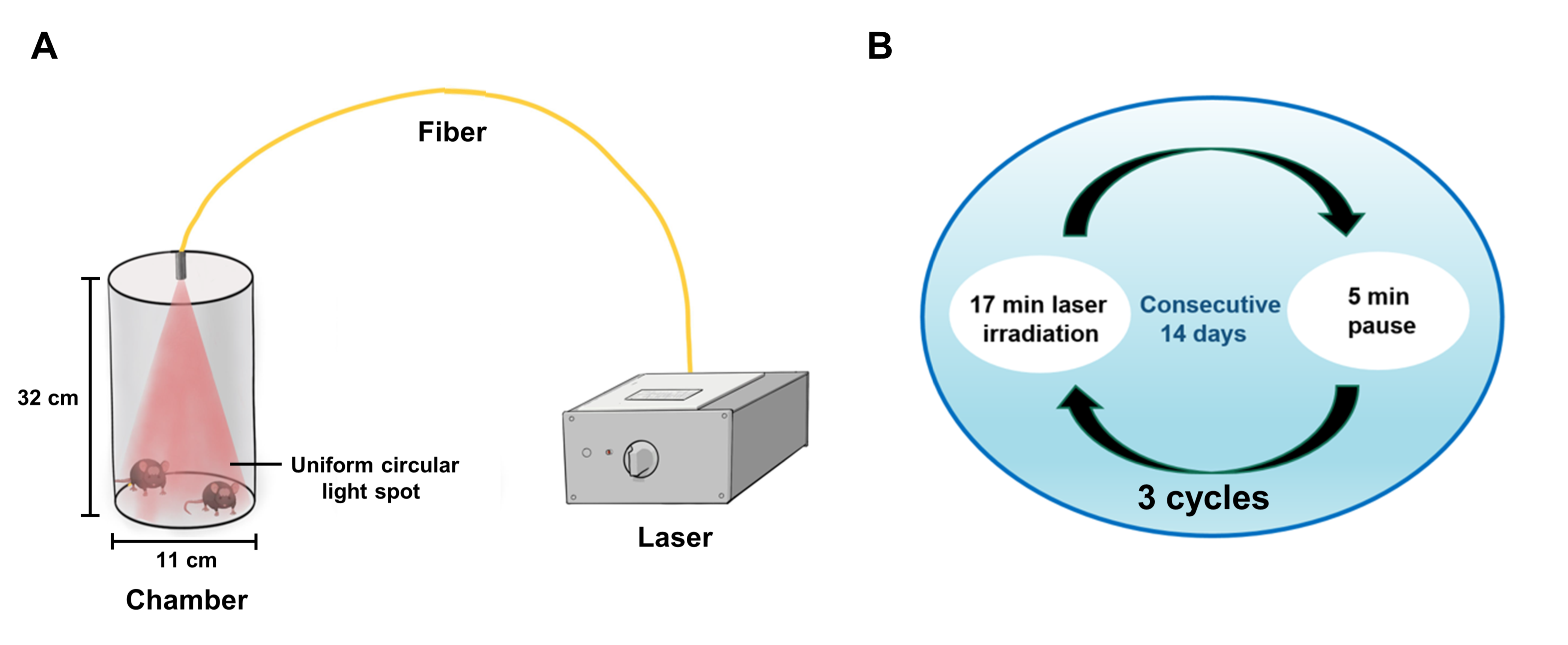


**Figure S1.** Experimental design. (A) The 1275-nm laser irradiation was performed in a chamber where mice with shaved heads were allowed to move freely. (B) A daily PBM during 2 weeks was carried out in sequence: 17-min laser irradiation followed by 5-min pause repeated three cycles so that the total experiment time was 61 min.


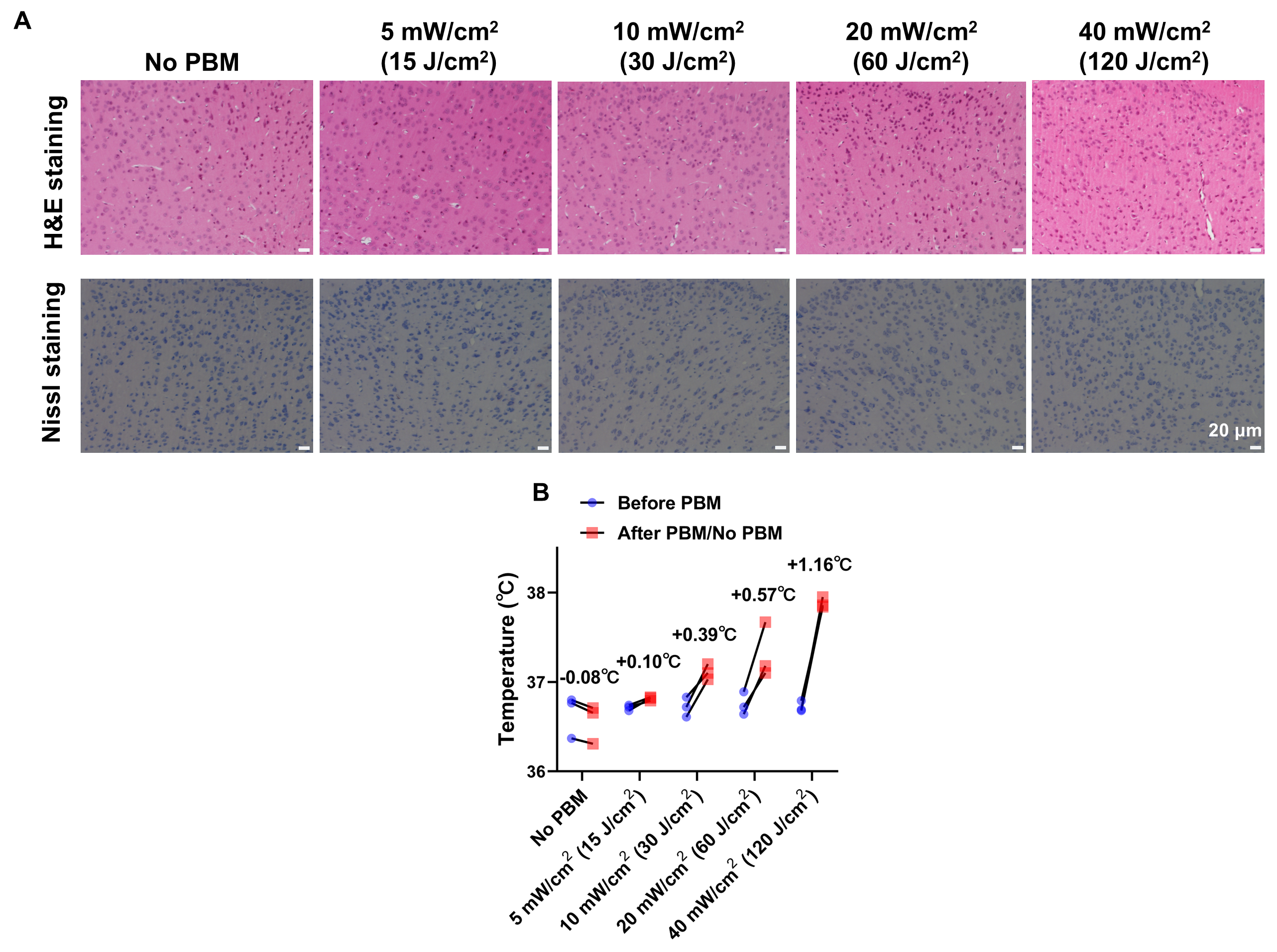


**Figure S2.** Safety assessment of PBM course with different laser power densities (energy densities). (A) Representative images of hematoxylin and eosin staining, as well as Nissl staining in the cortex after PBM course with different laser power densities (energy densities). Scale bar: 20 μm. (B) Measurement of temperature change on cortical surface of mice during a single PBM session with different power densities (energy densities).


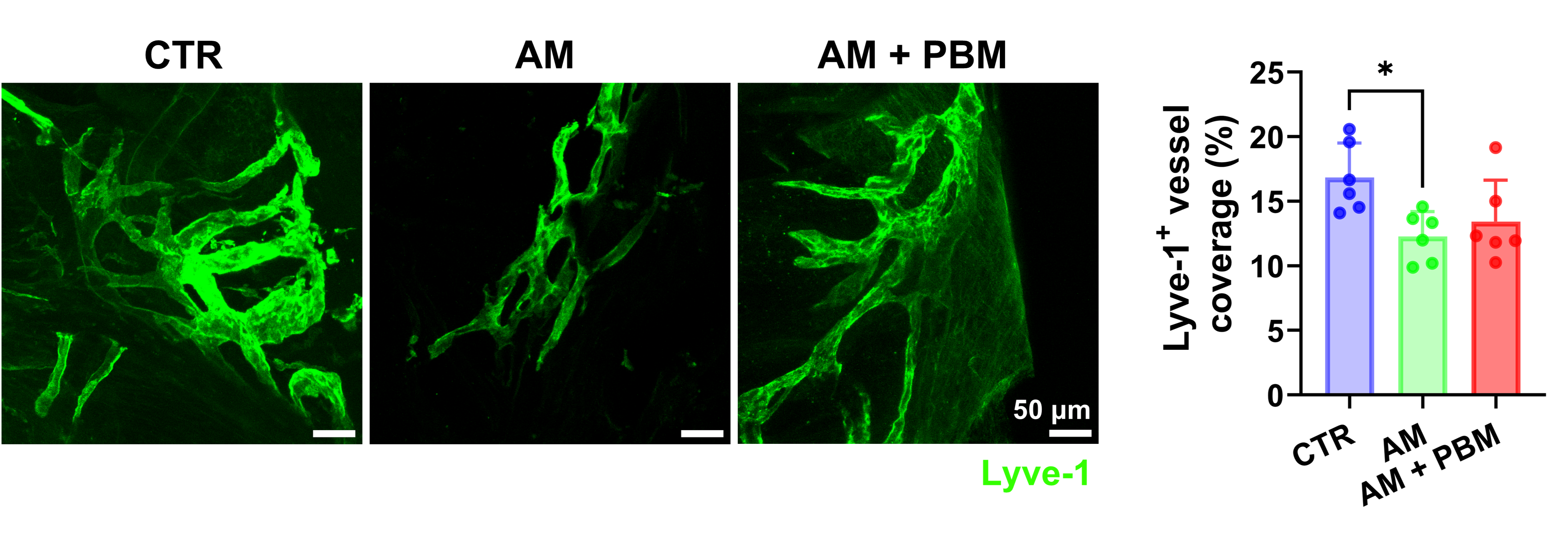


**Figure S3**. Effects of PBM course on MLVs network. Representative fluorescent images and quantitative analysis of the MLV coverage area around PSS in the groups of CTR, AM, and AM+PBM. Scale bar: 50 μm. At least two fields of view from each meningeal sample were used for the statistical analysis. The data are presented as mean ± standard deviation; *n* = 6 mice in each group; **p* < 0.05.

**
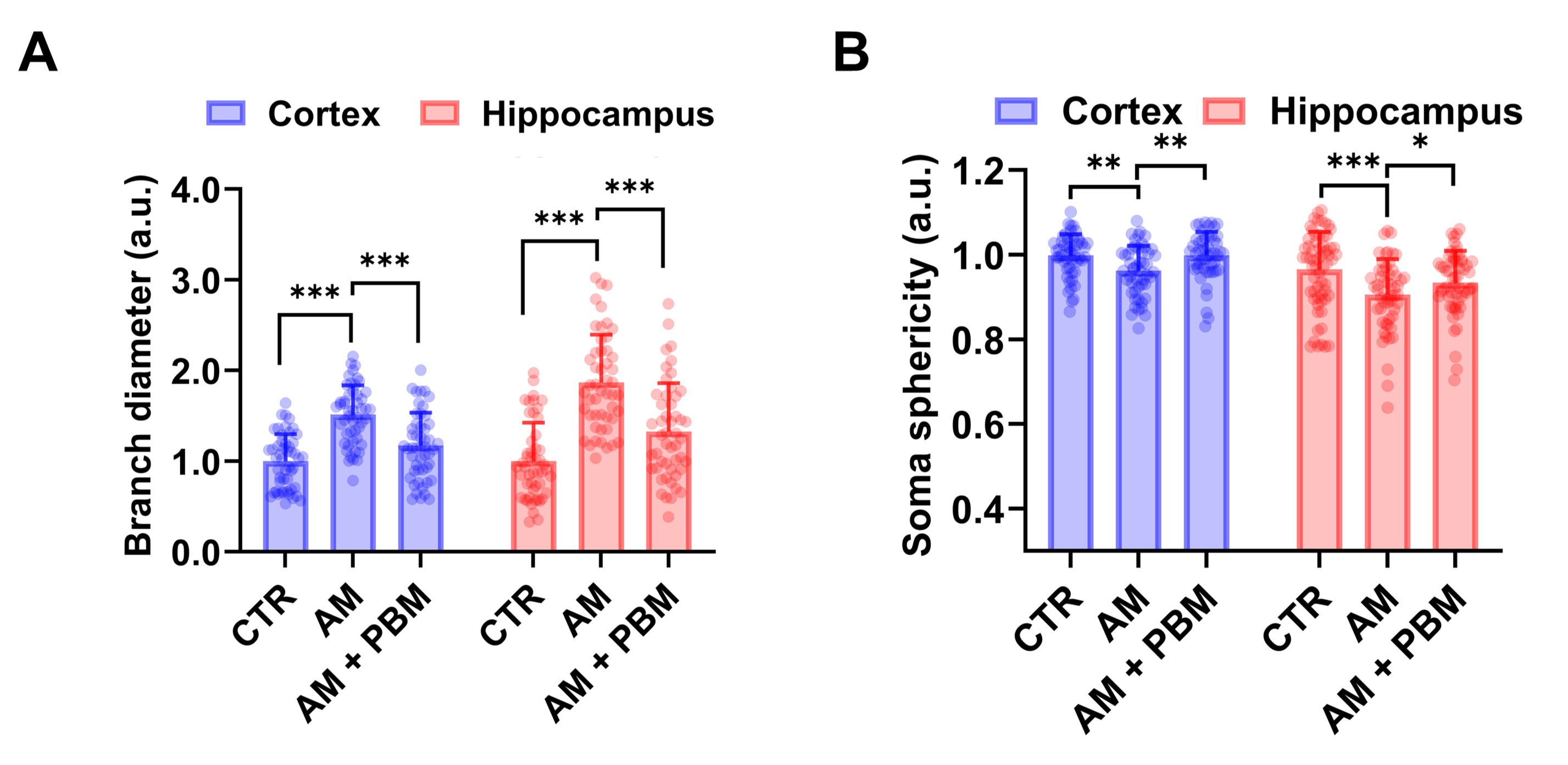
**

**Figure S4.** PBM course reduces the reactivity of microglia. (A) Quantitative analysis of the microglial branch diameter among CTR, AM, and AM+PBM groups. (B) Quantitative analysis of the microglial soma sphericity among three groups. Data in (A, B) are presented as mean ± standard deviation. *n* = 50 cells from five mice per group for A, B. **p* < 0.05, ***p* < 0.01, ****p* < 0.001.


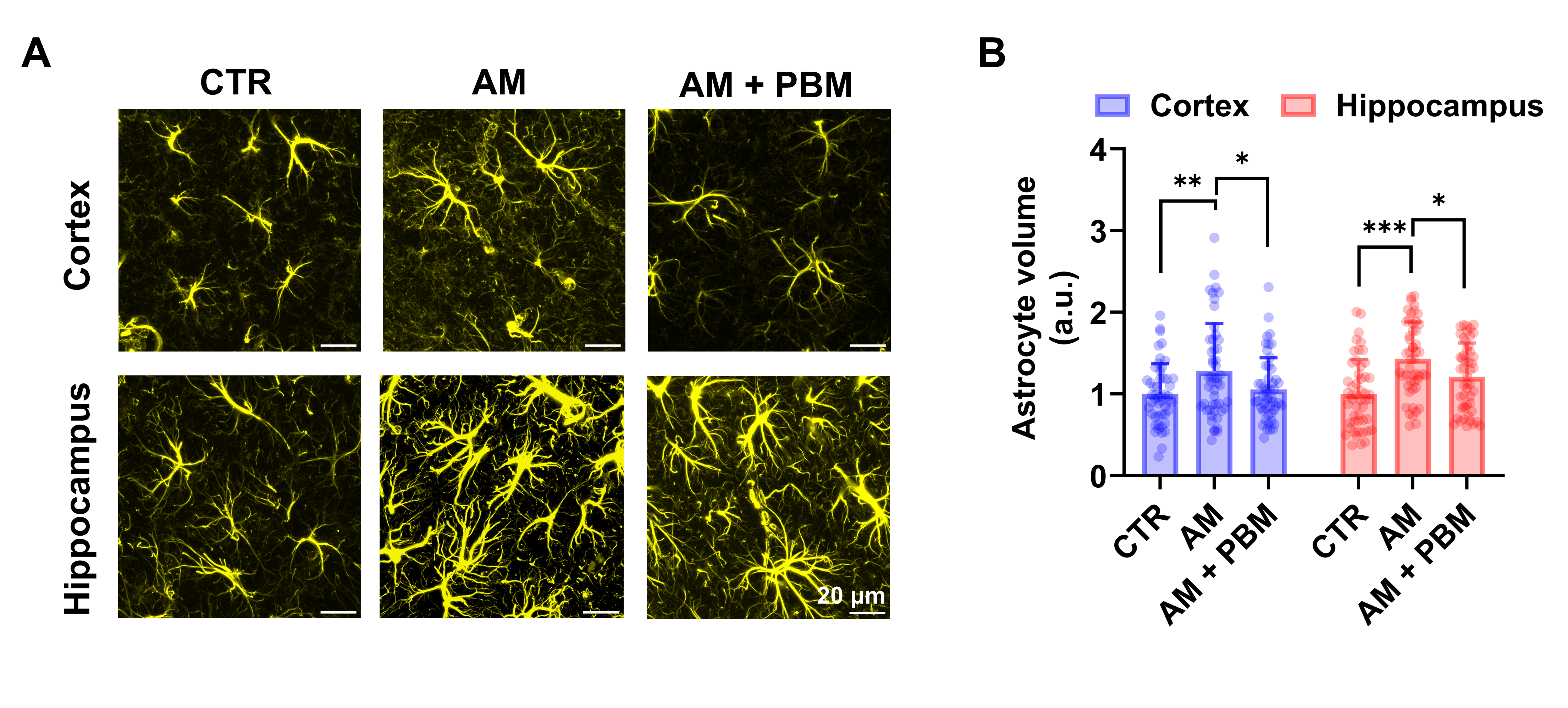


**Figure S5.** PBM course alleviates hypertrophy of astrocytes. (A) Representative images of the cortical and hippocampal astrocytes in the CTR, AM, and AM+PBM groups. Scale bar: 20 μm. (B) Quantitative analysis of the astrocytes volume among three groups. Data in (B) are presented as mean ± standard deviation. *n* = 50 cells from five mice per group for B. **p* < 0.05, ***p* < 0.01, ****p* < 0.001.


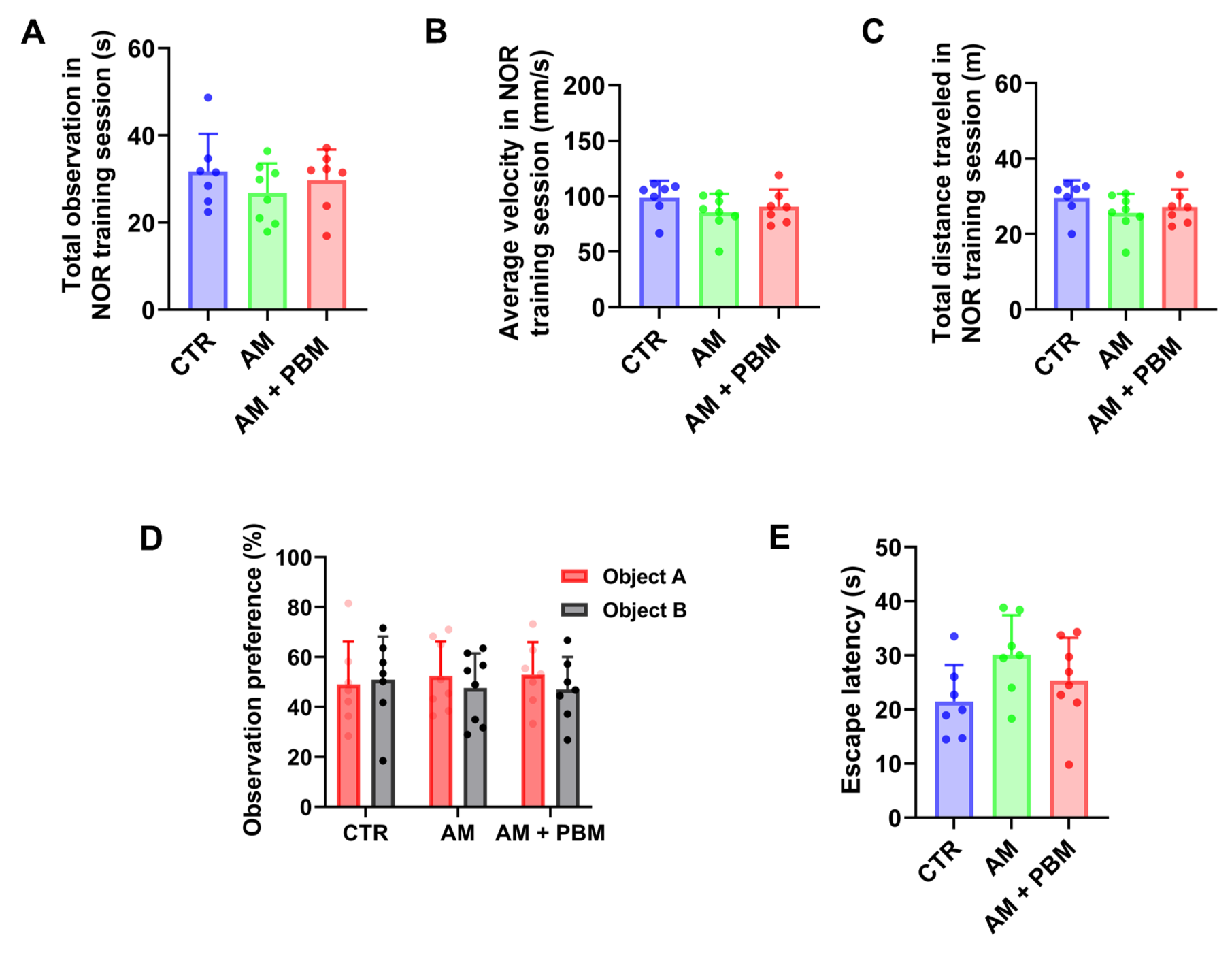


**Figure S6.** Effects of PBM course on the changes of motor activity and object preference in NOR training session and cognitive function in MWM probe trial. (A-C) Quantitative analysis of total observation time (A), average velocity (B), and total distance traveled (C) in NOR training session. (D) Quantitative analysis of object preference in NOR training session. (E) Quantitative analysis of escape latency in probe trial on Day 6 in MWM test. Data are presented as mean ± standard deviation, *n* = 7-8 mice per group.

**TABLE S1** Key resources.

| **REAGENT or RESOURCE** | **SOURCE** | **IDENTIFIER** |
| --- | --- | --- |
| **Antibodies** | | |
| anti-lyve-1 | R&D Systems | FAB2125G |
| anti-MBP | Proteintech | 15089-1-AP |
| anti-GFAP | EMD Millipore | MAB360 |
| anti-AGEs | Abcam | ab23722 |
| anti-caspase-3 | Proteintech | 19677-1-AP |
| anti-Mouse IgG (H + L) (Alexa Fluor 555) | Invitrogen | A-21422 |
| anti-Rabbit IgG (H + L) (Alexa Fluor 555) | Invitrogen | A-21429 |
| anti-Rabbit IgG (H + L) (Alexa Fluor 647) | Invitrogen | A-27040 |
| **Chemicals** | | |
| D-Galactose | Sigma | G0750 |
| FITC-Dextran | Sigma | 60842-46-8 |
| Evans blue dye | Sigma | E2129 |
| L-NAME | Sigma | N5751 |
| DAF-FM DA fluorescence probe | Beyotime | S0019S |
| DAPI | Invitrogen | D1306 |
| Hematoxylin and eosin | Solarbio | G1120 |
| Cresyl violet | Solarbio | G1430 |
| **Assay kits** | | |
| TUNEL assay kit | Elabscience | E-CK-A320 |
| ROS assay kit | Elabscience | E-BC-K138-F |
| MDA assay kit | Elabscience | E-BC-F007 |
| NO assay kit | Elabscience | E-BC-K135-M |
| SOD assay kit | Elabscience | E-BC-K020-M |
| BCA protein colorimetric assay kit | Elabscience | E-BC-K318-M |
| Mouse IL-1 beta ELISA Kit | ABclonal | RK00006 |
| Mouse IL-6 ELISA Kit | ABclonal | RK00008 |
| Mouse IL-4 ELISA Kit | ABclonal | RK00036 |
| Mouse IL-10 ELISA Kit | ABclonal | RK00016 |
